# Geographic variation in cardiometabolic risk factor prevalence explained by area-level disadvantage in the Illawarra-Shoalhaven region of the NSW, Australia

**DOI:** 10.1038/s41598-020-69552-4

**Published:** 2020-07-29

**Authors:** Renin Toms, Darren J. Mayne, Xiaoqi Feng, Andrew Bonney

**Affiliations:** 1 School of Medicine, University of Wollongong, Wollongong, NSW 2522 Australia; 2 Illawarra Health and Medical Research Institute, Wollongong, NSW 2522 Australia; 3 Public Health Unit, Illawarra Shoalhaven Local Health District, Warrawong, NSW 2502 Australia; 4 School of Public Health, The University of Sydney, Sydney, NSW 2006 Australia; 5 Population Wellbeing and Environment Research Lab (PowerLab), School of Health and Society, Faculty of Social Sciences, University of Wollongong, Wollongong, Australia; 6 School of Public Health and Community Medicine, University of New South Wales, Sydney, NSW Australia

**Keywords:** Disease prevention, Epidemiology, Risk factors

## Abstract

Cardiometabolic risk factors (CMRFs) demonstrate significant geographic variation in their distribution. The study aims to quantify the general contextual effect of the areas on CMRFs; and the geographic variation explained by area-level socioeconomic disadvantage. A cross sectional design and multilevel logistic regression methods were adopted. Data included objectively measured routine pathology test data between years 2012 and 2017 on: fasting blood sugar level; glycated haemoglobin; total cholesterol; high density lipoprotein; urinary albumin creatinine ratio; estimated glomerular filtration rate; and body mass index. The 2011 Australian census based Index of Relative Socioeconomic Disadvantage (IRSD) were the area-level study variables, analysed at its smallest geographic unit of reporting. A total of 1,132,029 CMRF test results from 256,525 individuals were analysed. After adjusting for individual-level covariates, all CMRFs significantly associated with IRSD and the probability of *higher risk* CMRFs increases with greater area-level disadvantage. Though the specific contribution of IRSD in the geographic variation of CMRF ranged between 57.8 and 14.71%, the general contextual effect of areas were found minimal (ICCs 0.6–3.4%). The results support universal interventions proportional to the need and disadvantage level of populations for the prevention and control of CMRFs, rather than any area specific interventions as the contextual effects were found minimal in the study region.

## Introduction

The prevalence of cardiometabolic risk factors (CMRFs) varies geographically^1,2^. Previous research has reported higher prevalence of CMRFs in certain localities: typically in areas of higher socioeconomic disadvantage^3–23^. However, frequently these studies have been based on measures of association or geographical variance rather than reporting clustering or the share of the total variance that is at the area-level^3–23^. Quantifying the clustering and the proportion of geographic variation in CMRFs contributed by area-level socioeconomic disadvantage can aid in designing appropriate area-level approaches to help prevent CMRFs. Chronic and uncontrolled CMRFs predispose individuals to the development of cardiovascular disease (CVD), which continues to be the leading cause of health care expenditure and premature mortality worldwide^24^.

In Australia, a social gradient is observed in the prevalence of many chronic conditions including various CMRFs (e.g.diabetes and chronic kidney disease)^25^. Generally, Australians enjoy better health than people in many other countries in the world. However, within Australia this better health is not equally distributed^26^. It is often-reported that socioeconomically disadvantaged individuals in Australia, on average, experience a greater disease burden than their less disadvantaged counterparts^25–28^. This tendency is also evident at a contextual level when studies have investigated association of CMRFs with area-level socioeconomic disadvantage in Australia ^5,7^ and globally ^4,9,10,12,14,16–18,20,23,29–33^.

Consistent with this, men from highly urbanised environments have been reported to have higher incidence of coronary heart disease with increasing residential area socioeconomic disadvantage, after adjusting for individual characteristics^18^. Also, lower area-level disadvantage has been reported as being associated with lower prevalence of some behavioural cardiac risk factors such as smoking, physical inactivity and obesity etc. in some studies^9,10,34^. Most of the reported associations of CMRFs with area-level socioeconomic disadvantage were independent of individual-level characteristics such as age and educational attainment. Even though the area-level associations of CMRFs were significant in these studies, the results were often dependent on the CMRF analysed, the measures of area-level socioeconomic disadvantage and the geographic scale at which associations were examined^35^.

Multilevel analyses of CMRFs based on the average measures of association or variation alone are insufficient to report the geographical variance as similar associations were possible with very different scenarios of area variance^36^. Multilevel findings extending on the general contextual effects and reporting the proportion of the total area-level variance along with the measures of clustering and the average measures of association or variation are appropriate and informative in reporting area-level influences, but less common^23,36–38^. To differentiate the relative importance of individual versus area-level interventions for the prevention and control of CMRFs, the geographical component of the total individual risk variance has to be identified in a multilevel approach.

Therefore, the aims of this study are to (1) quantify the general contextual or geographic effect of areas on CMRFs, over and above their individual-level compositions; and to (2) quantify the geographic variation across multiple CMRFs specifically explained by area-level socioeconomic disadvantage, within the Illawarra-Shoalhaven region of NSW Australia. Quantification of the general contextual effect and the variation specifically explained by area-level socioeconomic disadvantage will assist our understanding of the socioeconomic context of CMRFs in the study region and provide guidance for health service commissioning more generally nationally.

## Methods

A cross-sectional multilevel design was adopted to account for the hierarchical nature of the data and analyses. No informed consent were obtained for the individual-level data used in this study, as the study used existing data which were already de-identified. The study was approved by the University of Wollongong and Illawarra and Shoalhaven Local Health District Health and Medical Human Research Ethics Committee (HREC protocol No: 2017/124). All the methods and analyses were performed meeting the relevent ethical guidelines and regulations of the committee.

### Study area and data

The study was conducted in the Illawarra-Shoalhaven region of the New South Wales (NSW) state in Australia. The Illawarra-Shoalhaven region is a coastal plain along the south-east border of NSW; situates at the immediate south of the metropolitan boundaries of Sydney; and encompasses multiple regional cities, towns and rural areas. This region covers a land area of 5,615 km^2^, and had an estimated residential population of 369,469 at the time of the 2011 Australian Census of Population and Housing conducted by the Australian Bureau of Statistics (ABS)^39^. Statistical Area level 1 (SA1), the smallest geographical unit of the 2011 census data release, was the area-level unit of analysis in this study^39^. SA1s typically have a population size of 200 to 800 persons (average 400), and the Illawarra-Shoalhaven region covers a total of 980 conterminous SA1s^39^.

The CMRF test data in this study were extracted from the Southern IML Research (SIMLR) Study database, which is comprised of de-identified and internally linked pathology results from a major network of pathology services in the study region. The individual-level data in SIMLR database are geocoded to their corresponding SA1 areas, but not to their residential address, for privacy and confidentiality concerns. More details on this data source, procurement and access are published elsewhere^7^. The CMRF test data were extracted for non-pregnant individuals aged 18 years or older presenting for testing between 01 January 2012 and December 2017. Only the most recent test result was included if an individual had undergone the same test multiple times in this data period. Test data with missing details on the individual and area-level factors analysed in this study were excluded from the analyses.

### Variables

#### Outcome variable

Results of the CMRF tests were the individual-level outcome variables. Data on the seven CMRF tests analysed in this study included: fasting blood sugar level (FBSL); glycated haemoglobin (HbA1c); total cholesterol (TC); high density lipoprotein (HDL); urinary albumin creatinine ratio (ACR); estimated glomerular filtration rate (eGFR); and objectively-measured body mass index (BMI). These CMRF test results were dichotomised into *higher risk* and *lower risk* values based on the current national and international guidelines on risk definitions (Table 1).

**Table 1:** Definitions of *higher risk* CMRFs test results.

	*Higher risk* CMRFs	Definition
1	High FBSL	FBSL ≥ 7.0 mmol/L^40^
2	High HbA1c	HbA1c > 7.5% ^40^
3	High TC	TC ≥ 5.5 mmol/L ^41^
4	Low HDL	HDL < 1 mmol/L ^42^
5	High ACR	ACR ≥ 30 mcg/L to mg/L ^43^
6	Low eGFR	eGFR < 60 mL/min/1.73 m^2^ ^43^
7	Obesity	BMI ≥ 30 kg/m^2^ ^44^

*CMRFs* Cardiometabolic risk factors, *FBSL* Fasting Blood Sugar Level, *HbA1c* Glycated Haemoglobin, *TC* Total Cholesterol, *HDL* High Density Lipoprotein, *ACR* Albumin Creatinine Ratio, *eGFR* estimated Glomerular Filtration Rate, *BMI* Body Mass Index.

#### Study variable

The 2011 ABS census based Index of Relative Socioeconomic Disadvantage (IRSD) of the SA1s was the study variable. IRSD summarises a range of measures of relative socioeconomic disadvantage of people and households within SA1s and includes: level of income; education; employment; family structure; disability; housing; transportation; and internet connection^45^. This study uses IRSD reported as quintiles; the lowest quintile (Q1) indicating the most disadvantaged SA1s and the highest quintile (Q5) the least disadvantaged SA1s^45^. The IRSD quintiles in the study were derived by ABS from the distribution of IRSD scores for the Illawarra-Shoalhaven region based on the 2011 census. The study region has a diverse IRSD profile with representation across IRSD scores in comparison with Australia as a whole, making the region useful for population-level studies^46^.

#### Covariates

Analyses were adjusted for sex (male and female) and age group (18–29 years, 30–39 years, 40–49 years, 50–59 years, 60–69 years, 70–79 years, 80+ years) of each individual at the time of the pathology collection of the CMRFs tests analysed in his study.

### Statistical analyses

Initially, descriptive statistics of all individual and area-level variables were performed. Thereafter, single level and multilevel logistic regression models were fitted for the CMRF test data of individuals (Level 1), nested within SA1s (Level 2). For each of the seven CMRFs analysed in this study, a hierarchy of four multilevel models at SA1 level were fit that included fixed effects for age, sex and IRSD and random effect (intercept) for SA1. Model 0 was a single level model adjusted for age and sex; Model 1 (M1) was null model at level 2; Model 2 (M2) adjusted for age and sex at level 2; Model 3 (M3) adjusted for the area-level study variable (IRSD) only at level 2; and the final model Model 4 (M4) included both M2 and M3 covariates (age, sex and IRSD) at level 2. The estimated regression coefficients of the derived models were exponentiated to calculate odds ratios (ORs).The goodness of fit of the models were identified using Likelihood Ratio Tests (LRT) at *p* < 0.05 level of significance. The general equation of the fully adjusted model is:

1 $${\text{y}}_{{{\text{ij}} }} \sim {\text{Binomial }}\left( {{1}, \, \pi_{{{\text{ij}}}} } \right)$$

2 $${\text{logit}}\left( {\pi_{{{\text{ij}}}} } \right) = \beta_{0} + \beta_{{1}} {\text{Age}}_{{{\text{ij}}}} + \beta_{{2}} {\text{Sex}}_{{{\text{ij}} }} + \beta_{{3}} {\text{IRSD}}_{{{\text{ij}}}} {\text{ } + \text{ u}}_{{\text{j}}}$$

3 $${\text{u}}_{{{\text{j}} }} \sim {\text{N}}\left( {0, \, \tau^{{2}}_{{\text{u}}} } \right)$$

where *y*_*ij*_ denote the binary response of CMRF test outcome (as ‘*higher risk*’ or ‘*lower risk*’, based on the adopted definitions) for individual *i* in the area (SA1) *j*; *π*_*ij*_ denotes the probability that individual *i* in area (SA1) *j* has a ‘*higher risk*’ CMRF test outcome given their individual-level *age*_*ij*_ and *sex*_*ij*;_ and their area-level IRSD index. The *β*_1_*, β*_2_*, β*_3_ are the regression coefficients which measure the associations between the log-odds of the CMRF outcome and each covariate all else equal, and when exponentiated these are translated to ORs^36^. *u*_*j*_ is the random effect for the area (SA1) *j* and *τ*^2^_*u*_ is the area level variance, which has to be estimated.

### Model comparison

The Akaike Information Criterion (AIC) was used to evaluate model fit. The derived multilevel models were compared for: area-level variance (${\tau }^{2})$ at SA1 (level 2) level; proportional change in variance (PCV); Intra-cluster Correlation Coefficients (ICC); Median Odds Ratios (MORs); area under the receiver operating characteristic (AUC) curve; and the change in AUC.

The ${\tau }^{2}s$ of the multilevel models were initially identified from each models. PCVs were calculated for models M2s to M4s relative to M1s. The ICCs of the fitted models were calculated using the latent variable approach^47^. This approach assumes that a latent continuous outcome underlies the observed dichotomous outcomes and it is this latent outcome for which the ICC is calculated and interpreted. The ICC measured the expected correlation in CMRF outcomes between two individuals from the same SA1. The higher the ICC, the more relevant area-level context is for understanding individual latent outcome variation^36^. The MOR is calculated as an alternative way of interpreting the magnitude of area-level variance. The MOR translated the area-level variance which were estimated on the log-odds scale to the commonly used OR scale. The MOR result value is interpreted as the median increased odds of identifying the outcome if an individual move to another SA1 with higher risk. Thus, the higher the MOR the greater the general area-level effect and it will equal to 1 in the absence of area-level variance^36^. The general contextual effect of the geographic areas over and above their individual-level composition of the *higher risk* CMRFs, is obtained through the measure of clustering (ICC) in M2s. The geographic variance and ICC in the null models (M1s) of *higher risk* CMRFs may depend on both the contextual and individual-level variables. Therefore, M2s of the *higher risk* CMRFs which adjusted for individual-level attributes is better to provide information on the ‘general contextual effect’ of the areas. The unique contribution of the area-level study variable (IRSD) to the area-level variance of *higher risk* CMRFs were assessed through the PCVs between M2s and M4s.

The receiver operating characteristic (ROC) curves are created by plotting the true positive rates (TPR) i.e. sensitivity, against the false positive rates (FPR), i.e. 1 specificity for different binary classification thresholds of the predicted probabilities in all the models^48^. Post-estimation, predicted probabilities (π_ij_) are calculated for each individual and are used to calculate the AUC for the model. The AUCs of the models measure the capacity of the models to correctly classify individuals with or without the outcome of a *higher risk* CMRFs analysed in this study, as a function of their predicted probabilities^36^. The AUC values range from 1 and 0.5, where 1 is the perfect predictive discrimination and 0.5 have no predictive power^49^. The AUCs also indicate the general contextual effects and can be compared it to the ICC and the MOR values^36^. The added value of knowing an individual’s area of residence besides individual-level information (age and sex) can be obtained through the AUC change in Model 2 in reference to Model 0, where a higher AUC change would indicate higher relevance of areas in relation to CMRFs.

#### Statistical package

All analyses were performed using R version 3.4.4. (R Foundation for Statistical Computing, Vienna, Austria)^50^. Multi-level models were fit using the *glmer* function in the *lme4* package^51^^,^likelihood ratio tests were calculated using the *lrtest* function in the *lmtest* package^52^^,^and ROC curves using the *roc* function in the pROC package^53^.

## Results

**Figure 1:**
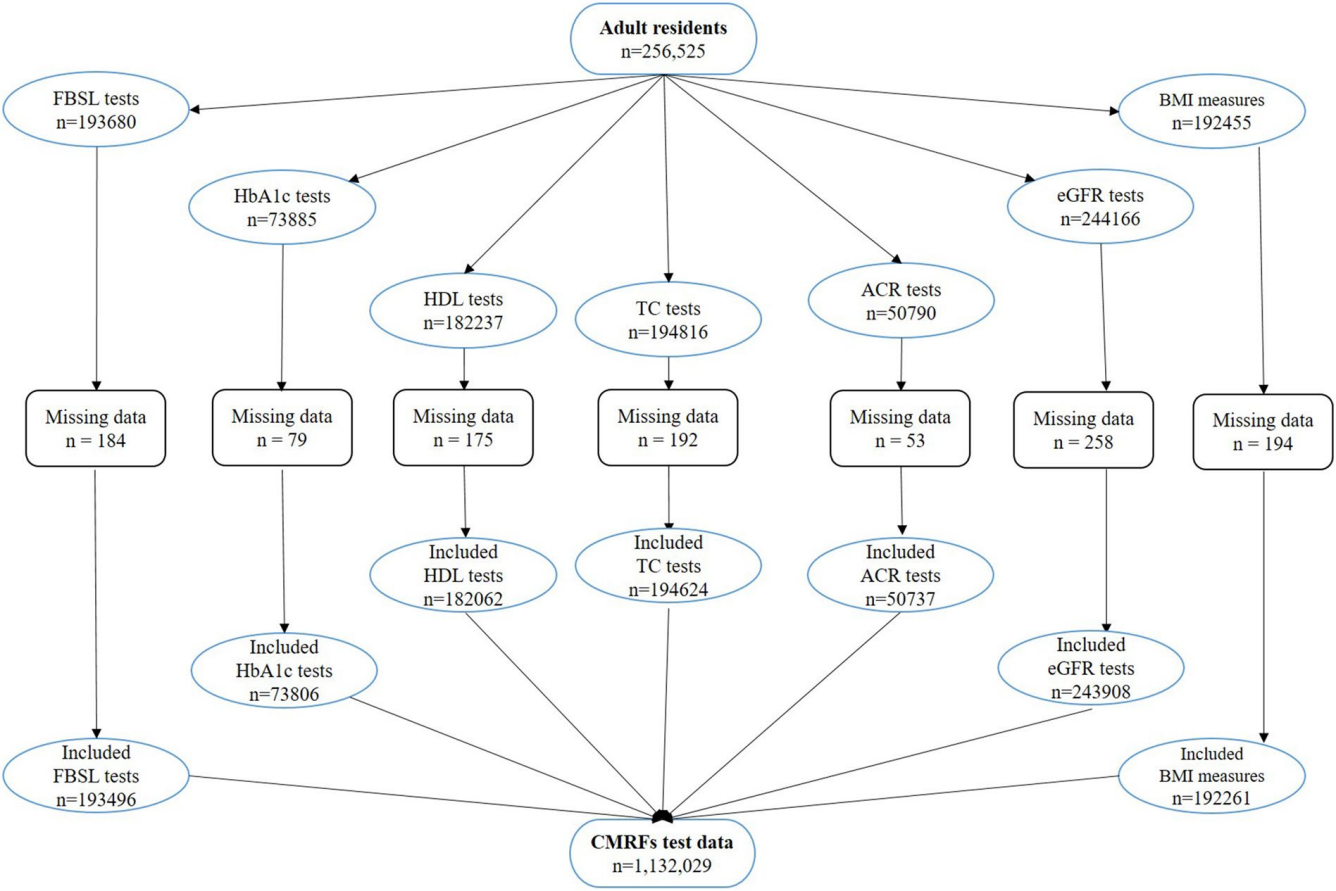
Flow chart of the included/excluded tests in the CMRFs test data.

A total of 1,132,029 CMRFs test data which belong to 256,525 individuals were extracted for the analyses. Figure 1 provides a flow chart of the individual tests in CMRF test data. The mean number of tests per person was 4.4. After removing 1,162 (1.0%) test results data with missing details, a total of 1,130,894 tests were included in the analytic data set.

Table 2 provides details of the missing data and test data distribution of each CMRF tests. Most frequently missing data were the IRSD indices from SA1s in the study area for which an IRSD index was not available from ABS 2011 census either due to low populations or poor data quality^54^.

**Table 2:** Table of excluded test data which had missing details.

	FBSL	HbA1c	TC	HDL	ACR	eGFR	BMI	Total
Extracted	193,680	73,885	194,816	182,237	50,790	244,166	192,455	1,132,029
**Missing data**								
Test value	1	0	0	0	0	0	0	1
Age	1	1	1	1	0	2	1	7
Sex	0	0	0	0	0	0	0	0
IRSD	182	78	191	174	53	256	193	1,154
Excluded tests	184	79	192	175	53	258	194	1,162
**Included tests**								
Total n	193,496	73,806	194,624	182,062	50,737	243,908	192,261	1,130,894
(%)	(17.11)	(6.53)	(17.21)	(16.10)	(4.49)	(21.57	(17.00)	(100.00)

*FBSL* Fasting Blood Sugar Level, *HbA1c* Glycated Haemoglobin, *TC* Total Cholesterol, *HDL* High Density Lipoprotein, *ACR* Albumin Creatinine Ratio, *eGFR* estimated Glomerular Filtration Rate, *BMI* Body Mass Index, *IRSD* Index of the Relative Socioeconomic Disadvantage, *SA1* Statistical Area level 1.

Tables 3 and 4 shows the frequencies and relative frequencies of CMRF tests results. Overall, the *higher risk* frequencies of all CMRFs increased with increasing area-level socioeconomic disadvantage, except for TC which demonstrated an inverse trend.

**Table 3:** Cross-tabulation of individual CMRFs (FBSL, HbA1c, TC and HDL) with the variables in study.

CMRFs	FBSL		HbA1c		TC		HDL	
	Total tests	Higher risk* results, n (%)	Total tests	Higher risk* results, n (%)	Total tests	Higher risk* results, n (%)	Total tests	Higher risk* results, n (%)
Rates	193,496	16,259 (8.4)	73,806	7,920 (10.73)	194,624	57,506 (29.55)	182,062	21,238 (11.67)
**Sex**								
Male	83,603	9,279 (4.8)	35,757	4,444 (6.02)	90,950	23,503 (12.0)	85,266	15,872 (8.72)
Female	109,893	6,980 (3.6)	38,049	3,476 (4.71)	103,674	34,003 (17.47)	96,796	5,366 (2.95)
**Age (years)**								
18–29	19,747	238 (0.1)	3,480	250 ( 0.34)	14,247	2,127 (1.09)	11,435	1,377 (0.76)
30–39	23,515	459 (0.2)	4,889	293 ( 0.40)	18,960	4,889 (2.51)	16,787	2,301 (1.26)
40–49	29,424	1,265 (0.65)	8,447	760 ( 1.03)	31,395	10,719 (5.51)	29,339	3,585 (1.97)
50–59	37,085	2,948 (1.52)	13,510	1,507 (2.04)	39,663	16,316 (8.38)	37,824	4,283 (2.35)
60–69	37,962	4,670 (2.41)	17,665	2,064 (2.80)	40,471	13,620 (7.00)	39,134	4,227 (2.32)
70–79	29,009	4,396 (2.27)	15,715	1,860 (2.52)	31,186	6,748 (3.47)	30,114	3,419 (1.88)
80+	16,754	2,283 (1.18)	10,100	1,186 (1.61)	18,702	3,087 (1.59)	17,429	2046 (1.12)
**IRSD**								
Most D Q-1	38,885	4,495 (2.32)	17,024	2,429 (3.29)	39,347	10,631 (5.46)	36,625	5,520 (3.03)
Q-2	41,545	3,757 (1.94)	16,680	1,875 (2.54)	41,937	12,015 (6.17)	39,050	4,901 (2.69)
Q-3	39,828	3,386 (1.75)	15,376	1,585 (2.15)	40,401	12,045 (6.19)	37,794	4,201 (2.31)
Q-4	37,137	2,594 (1.34)	13,101	1,138 (1.54)	36,865	11,163 (5.74)	34,566	3,581 (1.97)
Least D Q-5	36,101	2027 (1.05)	11,625	893 (1.21)	36,074	11,652 (5.99)	34,027	3,035 (1.67)

*FBSL* Fasting Blood Sugar Level, *HbA1c* Glycated Haemoglobin, *TC* Total Cholesterol, *HDL* High Density Lipoprotein, *Most D* Most Disadvantaged, *Least D* Least Disadvantaged

*Refer to Table 1 for *high risk* threshold levels of CMRFs.

**Table 4:** Cross-tabulation of individual CMRFs (ACR, eGFR, and Obesity) with the variables in study.

CMRFs	ACR		eGFR		Obesity	
	Total tests	Higher risk* results, n (%)	Total tests	Higher risk* results, n (%)	Total tests	Higher risk* results, n (%)
Rates	50,737	2046 (4.03)	243,908	27,205 (11.15)	192,261	64,875 (33.7)
**Sex**						
Male	25,043	1,265 (2.49)	108,140	12,441 (5.1)	86,853	29,585 (15.3)
Female	25,694	781 (1.54)	135,768	14,764 (6.05)	105,408	35,290 (18.3)
**Age (years)**						
18–29	1546	47 (0.09)	32,961	72 (0.03)	23,277	4,582 (2.38)
30–39	2,278	71 (0.14)	29,047	105 (0.04)	22,799	6,535 (3.40)
40–49	4,870	108 (0.21)	35,778	330 (0.14)	30,401	10,595 (5.51)
50–59	9,272	230 (0.45)	42,695	1,112 (0.46)	37,285	13,825 (7.19)
60–69	13,388	412 (0.81)	43,423	3,626 (1.49)	38,370	15,310 (7.96)
70–79	12,337	605 (1.19)	34,406	8,507 (3.49)	30,074	11,324 (5.89)
80+	7,046	573 (1.13)	25,598	13,453 (5.52)	10,055	2,704 (1.41)
**IRSD**						
Most D Q-1	11,915	638 (1.26)	49,288	7,061 (2.89)	37,476	15,365 (7.99)
Q-2	11,350	485 (0.96)	52,947	6,354 (2.61)	40,172	14,334 (7.46)
Q-3	10,494	391 (0.77)	50,816	5,917 (2.43)	39,133	13,007 (6.77)
Q-4	8,732	308 (0.61)	46,440	4,406 (1.81)	37,370	11,766 (6.12)
Least D Q-5	8,246	224 (0.44)	44,417	3,467 (1.42)	38,110	10,403 (5.41)

ACR Albumin Creatinine Ratio, *eGFR* estimated Glomerular Filtration Rate, *BMI* Body Mass Index, *Most D* Most Disadvantaged, *Least D* Least Disadvantaged.

*Refer to Table 1 for *higher risk* threshold levels of CMRFs.

**Table 5:** Single and multilevel logistic regression model summaries for high FBSL (FBSL ≥ 7.0 mmol/L).

	Single level model	Multilevel models			
	Model 0	Model 1	Model 2	Model 3	Model 4
Significance (LRT)	***	***	***	***	***
High FBSL	OR (95% CI)	OR (95% CI)	OR (95% CI)OR (95% CI)	OR (95% CI)	OR (95% CI)
Intercept	0.01 (0.01–0.01)	0.09 (0.09–0.09)	0.01 (0.01–0.01)	0.06 (0.06–0.06)	0.01 (0.01–0.01)
**Sex**					
Female	Reference		–		
Male	1.62 (1.56–1.67)		1.63 (1.56–1.7)		1.63 (1.58–1.7)
**Age**					
18–29	Reference		–		1.64 (1.41–1.9)
30–39	1.60 (1.36–1.87)		1.63 (1.39–1.9)		3.58 (3.12–4.1)
40–49	3.41 (2.97–3.93)		3.53 (3.07–4.1)		6.81 (5.98–7.8)
50–59	6.48 (5.68–7.42)		6.77 (5.93–7.7)		11.05 (9.71–12.6)
60–69	10.48 (9.21–11.98)		11.07 (9.72–12.6)		13.74 (12.07–15.6)
70–79	13.35 (11.73–15.27)		13.93 (12.22–15.9)		12.02 (10.52–13.7)
80+	12.01 (10.51–13.78)		12.33 (10.79–14.1)		
**IRSD**					
Q-5				Reference	–
Q-4				1.27 (1.18–1.36)	1.27 (1.18–1.37)
Q-3				1.58 (1.47–1.69)	1.49 (1.39–1.61)
Q-2				1.68 (1.57–1.80)	1.62 (1.50–1.74)
Most D Q-1				2.20 (2.06–2.36)	2.11 (1.96–2.26)
**AIC**	103,645	111,022.8	103,066.2	110,552.5	102,689.6
**Variance**		0.101	0.103	0.034	0.044
**PCV**		–	+ 1.88%	− 66.41%	− 56.33%
**ICC (%)**		3.00	3.00	1.0	1.3
**MOR**		1.35	1.36	1.19	1.22
**AUC**	0.70	0.61	0.73	0.60	0.72
**AUC change**^†^			+ 0.03		
Proportional variance explained by IRSD: 57.14%					

***p < 0.001; ^†^Change in Model 2 in relation to Model 0; Model 0—Single level model adjusted for age + sex; Model 1—null model at SA1 level; Model 2—M1 + individual-level: age + sex; Model 3—Model 1 + Area level: IRSD quintiles of SA1s; Model 4—Model 1 + Model 2 + Model 3.

**Table 6:** Single and multilevel logistic regression model summaries for high HbA1c (HbA1c > 7.5%).

	Single level model	Multilevel models			
	Model 0	Model 1	Model 2	Model 3	Model 4
Significance (LRT)	***	***	***	***	***
High HbA1c	OR (95% CI)	OR (95% CI)	OR (95% CI)	OR (95% CI)	OR (95% CI)
Intercept	0.07 (0.06–0.08)	0.12 (0.12–0.12)	0.07 (0.06–0.07)	0.08 (0.08–0.09)	0.05 (0.04–0.05)
**Sex**					
Female	Reference		–		–
Male	1.37 (1.31–1.43)		1.38 (1.32–1.45)		1.39 (1.32–1.45)
**Age**					
18–29	Reference		–		–
30–39	0.81 (0.68–0.96)		0.81 (0.68–0.96)		0.81 (0.68–0.96)
40–49	1.22 (1.06–1.42)		1.24 (1.07–1.44)		1.26 (1.08–1.46)
50–59	1.53 (1.34–1.77)		1.56 (1.36–1.80)		1.57 (1.36–1.81)
60–69	1.61 (1.40–1.85)		1.64 (1.43–1.88)		1.64 (1.43–1.88)
70–79	1.63 (1.42–1.87)		1.64 (1.42–1.88)		1.62 (1.41–1.86)
80 +	1.64 (1.43–1.90)		1.63 (1.41–1.88)		1.60 (1.39–1.85)
**IRSD**					
Q-5				Reference	–
Q-4				0.08 (0.08–0.09)	1.15 (1.04–1.28)
Q-3				1.14 (1.03–1.27)	1.39 (1.26–1.54)
Q-2				1.40 (1.27–1.54)	1.55 (1.41–1.71)
Most D Q-1				1.54 (1.40–1.69)	2.02 (1.84–2.22)
**AIC**	49,897	50,114.5	49,690.2	49,875.3	49,453.3
**Variance**		0.103	0.106	0.047	0.049
**PCV**		–	+ 3.02%	− 54.82%	− 51.91%
**ICC (%)**		3.0	3.1	1.4	1.5
**MOR**		1.36	1.36	1.23	1.24
**AUC**	0.56	0.63	0.64	0.61	0.63
**AUC change**†			+ 0.08		
Proportional variance explained by IRSD: 53.31%					

***p < 0.001; ^†^Change in Model 2 in relation to Model 0; Model 0—Single level model adjusted for age + sex; Model 1—null model at SA1 level; Model 2—Model 1 + individual-level: age + sex; Model 3—Model 1 + Area level: IRSD quintiles of SA1s; Model 4—Model 1 + Model 2 + Model 3.

**Table 7:** Single and multilevel logistic regression model summaries for high TC (TC ≥ 5.5 mmol/L).

	Single level model	Multilevel models			
	Model 0	Model 1	Model 2	Model 3	Model 4
Significance (LRT)	***	***	***	***	***
High TC	OR (95% CI)	OR (95% CI)	OR (95% CI)	OR (95% CI)	OR (95% CI)
Intercept	0.20 (0.19–0.21)	0.42 (0.42–0.42)	0.20 (0.19–0.21)	0.08 (0.08–0.09)	0.22 (0.21–0.23)
**Sex**					
Female	Reference		–		–
Male	0.69 (0.68–0.71)		0.69 (0.68–0.71)		0.69 (0.68–0.71)
**Age**					
18–29	Reference		–		–
30–39	2.01 (1.90–2.13)		2.02 (1.91–2.14)		2.01 (1.90–2.13)
40–49	3.01 (2.86–3.17)		3.01 (2.86–3.17)		3.00 (2.85–3.16)
50–59	4.09 (3.89–4.30)		4.08 (3.88–4.29)		4.07 (3.87–4.28)
60–69	2.97 (2.83–3.13)		2.95 (2.80–3.10)		2.95 (2.80–3.10)
70–79	1.61 (1.53–1.70)		1.60 (1.52–1.69)		1.61 (1.52–1.70)
80 +	1.14 (1.07–1.21)		1.13 (1.07–1.20)		1.14 (1.07–1.21)
**IRSD**					
Q-5				Reference	–
Q-4				0.91 (0.87–0.95)	0.94 (0.90–0.98)
Q-3				0.88 (0.85–0.92)	0.94 (0.90–0.98)
Q-2				0.84 (0.80–0.87)	0.90 (0.87–0.94)
Most D Q-1				0.77 (0.74–0.81)	0.84 (0.81–0.88)
**AIC**	227,464	235,931.6	227,254.6	235,795.4	227,199.2
**Variance**		0.026	0.020	0.018	0.017
**PCV**		-	− 21.76%	− 27.81%	− 33.27%
**ICC (%)**		0.8	0.6	0.6	0.5
**MOR**		1.16	1.14	1.14	1.13
**AUC**	0.63	0.56	0.64	0.56	0.64
**AUC change**^†^			+ 0.01		
Proportional variance explained by IRSD: 14.71%					

***p < 0.001; ^†^Change in Model 2 in relation to Model 0; Model 0—Single level model adjusted for age + sex; Model 1—null model at SA1 level; Model 2—M1 + individual-level: age + sex; Model 3—Model 1 + Area level: IRSD quintiles of SA1s; Model 4—Model 1 + Model 2 + Model 3.

**Table 8:** Single and multilevel logistic regression model summaries for low HDL (HDL < 1 mmol/L).

	Single level model	Multilevel models			
	Model 0	Model 1	Model 2	Model 3	Model 4
Significance (LRT)	***	***	***	***	***
Low HDL	OR (95% CI)	OR (95% CI)	OR (95% CI)	OR (95% CI)	OR (95% CI)
Intercept	0.06 (0.06–0.07)	0.13 (0.13–0.13)	0.06 (0.06–0.07)	0.10 (0.09–0.10)	0.05 (0.04–0.05)
**Sex**					
Female	Reference		–		–
Male	3.92 (3.80–4.05)		3.98 (3.85–4.11)		3.98 (3.85–4.11)
**Age**					
18–29	Reference		–		–
30–39	1.11 (1.03–1.20)		1.11 (1.03–1.20)		1.12 (1.04–1.21)
40–49	0.97 (0.91–1.04)		0.99 (0.92–1.05)		1.00 (0.93–1.07)
50–59	0.87 (0.81–0.93)		0.88 (0.82–0.94)		0.89 (0.83–0.95)
60–69	0.81 (0.76–0.87)		0.82 (0.77–0.88)		0.82 (0.77–0.88)
70–79	0.85 (0.80–0.91)		0.86 (0.80–0.92)		0.85 (0.79–0.91)
80+	0.94 (0.87–1.01)		0.93 (0.86–1.00)		0.91 (0.85–0.98)
**IRSD**					
Q-5				Reference	–
Q-4				1.18 (1.11–1.26)	1.20 (1.13–1.28)
Q-3				1.29 (1.21–1.37)	1.32 (1.24–1.41)
Q-2				1.48 (1.39–1.57)	1.51 (1.42–1.61)
Most D Q-1				1.81 (1.71–1.92)	1.90 (1.78–2.02)
**AIC**	123,277	130,649.7	122,700.0	130,294.3	122,328.3
**Variance**		0.071	0.081	0.030	0.034
**PCV**		–	+15.25%	−58.05%	−51.37%
**ICC (%)**		2.1	2.4	0.9	1.0
**MOR**		1.29	1.31	1.18	1.19
**AUC**	0.67	0.60	0.71	0.59	0.70
**AUC change**^†^			+ 0.04		
Proportional variance explained by IRSD: 57.8%					

***p < 0.001; ^†^Change in Model 2 in relation to Model 0; Model 0—Single level model adjusted for age + sex; Model 1—null model at SA1 level; Model 2—M1 + individual-level: age + sex; Model 3—Model 1 + Area level: IRSD quintiles of SA1s; Model 4—Model 1 + Model 2 + Model 3.

**Table 9:** Single and multilevel logistic regression model summaries for high ACR (ACR ≥ 30 mcg/L to mg/L).

	Single level model	Multilevel models			
	Model 0	Model 1	Model 2	Model 3	Model 4
Significance (LRT)	***	***	***	***	***
High ACR	OR (95% CI)	OR (95% CI)	OR (95% CI)	OR (95% CI)	OR (95% CI)
Intercept	0.02 (0.02–0.03)	0.04 (0.04–0.04)	0.02 (0.02–0.03)	0.03 (0.02–0.03)	0.02 (0.01–0.02)
**Sex**					
Female	Reference	–	–		–
Male	1.74 (1.59–1.91)		1.75 (1.59–1.92)		1.76 (1.60–1.93)
**Age**					
18–29	Reference		–		–
30–39	0.99 (0.69–1.45)		1.00 (0.69–1.45)		0.99 (0.68–1.44)
40–49	0.68 (0.49–0.98)		0.69 (0.49–0.97)		0.70 (0.49–0.98)
50–59	0.76 (0.56–1.06)		0.77 (0.56–1.05)		0.77 (0.56–1.05)
60–69	0.95 (0.70–1.30)		0.95 (0.70–1.30)		0.95 (0.70–1.29)
70–79	1.54 (1.15–2.11)		1.55 (1.14–2.09)		1.52 (1.12–2.05)
80+	2.73 (2.04–3.74)		2.74 (2.02–3.71)		2.65 (1.96–3.59)
**IRSD**					
Q-5				Reference	–
Q-4				1.31 (1.10–1.57)	1.25 (1.04–1.50)
Q-3				1.39 (1.16–1.65)	1.27 (1.06–1.51)
Q-2				1.61 (1.36–1.90)	1.45 (1.23–1.72)
Most D Q-1				2.02 (1.72–2.38)	1.84 (1.56–2.16)
**AIC**	16,596	17,130.0	16,585.2	17,053.0	16,527.2
**Variance**		0.092	0.073	0.044	0.036
**PCV**		–	− 20.53%	− 52.88%	− 61.19%
**ICC (%)**		2.7	2.2	1.3	1.1
**MOR**		1.34	1.30	1.22	1.20
**AUC**	0.65	0.70	0.69	0.62	0.67
**AUC change**†			+ 0.04		
Proportional variance explained by IRSD: 51.17%					

***p < 0.001; ^†^Change in Model 2 in relation to Model 0; Model 0—Single level model adjusted for age + sex; Model 1—null model at SA1 level; Model 2—M1 + individual-level: age + sex; Model 3—Model 1 + Area level: IRSD quintiles of SA1s; Model 4—Model 1 + Model 2 + Model 3.

**Table 10:** Single and multilevel logistic regression model summaries for low eGFR (eGFR < 60 mL/min/1.73 m^2^).

	Single level model	Multilevel models			
	Model 0	Model 1	Model 2	Model 3	Model 4
Significance (LRT)	***	***	***	***	***
Low eGFR	OR (95% CI)	OR (95% CI)	OR (95% CI)	OR (95% CI)	OR (95% CI)
Intercept	0.00 (0.00–0.00)	0.13 (0.12–0.13)	0.00 (0.00–0.00)	0.08 (0.07–0.08)	0.00 (0.00–0.00)
**Sex**					
Female	Reference		–		–
Male	0.98 (0.95–1.01)		0.98 (0.95–1.01)		0.98 (0.95–1.01)
**Age**					
18–29	Reference		–		–
30–39	1.66 (1.23–2.25)		1.66 (1.24–2.20)		1.66 (1.24–2.22)
40–49	4.26 (3.32–5.54)		4.26 (3.34–5.42)		4.30 (3.36–5.49)
50–59	12.24 (9.72–15.68)		12.26 (9.78–15.35)		12.32 (9.80–15.47)
60–69	41.72 (33.30–53.19)		41.81 (33.55–52.1)		41.86 (33.49–52.31)
70–79	150.44 (120.24–191.57)		150.66 (121–187.6)		149.53 (120–186.6)
80+	506.80 (405.05–645.35)		509.18 (409–633.9)		501.47 (401.7–626)
**IRSD**					
Q-5				Reference	–
Q-4				1.23 (1.12–1.35)	1.09 (1.03–1.16)
Q-3				1.59 (1.45–1.74)	1.19 (1.13–1.26)
Q-2				1.65 (1.51–1.81)	1.22 (1.15–1.29)
Most D Q-1				1.97 (1.80–2.15)	1.38 (1.31–1.46)
**AIC**	115,340	167,164.8	115,257.1	166,930.0	115,125.7
**Variance**		0.189	0.024	0.138	0.014
**PCV**		–	− 87.26%	− 26.84%	− 92.79%
**ICC (%)**		5.4	0.7	4.0	0.4
**MOR**		1.51	1.16	1.43	1.12
**AUC**	0.88	0.64	0.89	0.63	0.88
**AUC change**†			+ 0.01		
Proportional variance explained by IRSD: 41.75%					

***p < 0.001; ^†^Change in Model 2 in relation to Model 0; Model 0—Single level model adjusted for age + sex; Model 1—null model at SA1 level; Model 2—M1 + individual-level: age + sex; Model 3—Model 1 + Area level: IRSD quintiles of SA1s; Model 4—Model 1 + Model 2 + Model 3.

**Table 11:** Single and multilevel logistic regression model summaries for obesity (BMI ≥ 30 kg/m^2^).

	Single level model	Multilevel models			
	Model 0	Model 1	Model 2	Model 3	Model 4
Significance (LRT)	***	***	***	***	***
Obesity	OR (95% CI)	OR (95% CI)	OR (95% CI)	OR (95% CI)	OR (95% CI)
Intercept	0.25 (0.24–0.25)	0.51 (0.50–0.51)	0.25 (0.24–0.26)	0.37 (0.35–0.39)	0.18 (0.17–0.19)
**Sex**					
Female	Reference		–		–
Male	0.99 (0.97–1.00)		0.99 (0.97–1.01)		0.99 (0.97–1.01)
**Age**					
18–29	Reference		–		–
30–39	1.64 (1.57–1.71)		1.63 (1.56–1.71)		1.64 (1.57–1.71)
40–49	2.18 (2.10–2.27)		2.20 (2.11–2.29)		2.21 (2.12–2.30)
50–59	2.41 (2.32–2.50)		2.44 (2.34–2.53)		2.44 (2.35–2.54)
60–69	2.71 (2.61–2.82)		2.73 (2.63–2.84)		2.74 (2.63–2.84)
70–79	2.47 (2.37–2.57)		2.44 (2.34–2.54)		2.43 (2.33–2.53)
80+	1.50 (1.42–1.59)		1.46 (1.38–1.55)		1.45 (1.37–1.53)
**IRSD**					
Q-5				Reference	-
Q-4				1.25 (1.17–1.33)	1.26 (1.18–1.34)
Q-3				1.37 (1.29–1.46)	1.38 (1.30–1.47)
Q-2				1.51 (1.42–1.61)	1.54 (1.44–1.64)
Most D Q-1				1.90 (1.79–2.03)	1.94 (1.83–2.07)
**AIC**	242,064	242,793.2	239,122.6	242,443.7	238,748.4
**Variance**		0.115	0.117	0.071	0.069
**PCV**		–	+ 1.48%	− 38.76%	− 40.30%
**ICC (%)**		3.4	3.4	2.1	2.0
**MOR**		1.38	1.39	1.29	1.28
**AUC**	0.56	0.60	0.63	0.60	0.62
**AUC change**^†^			+ 0.07		
Proportional variance explained by IRSD: 41.06%					

***p < 0.001; ^†^Change in Model 2 in relation to Model 0; Model 0—Single level model adjusted for age + sex; Model 1—Single level model adjusted for age + sex; Model 1—null model at SA1 level; Model 2—M1 + individual-level: age + sex; Model 3—Model 1 + Area level: IRSD quintiles of SA1s; Model 4—Model 1 + Model 2 + Model 3.

Single and multilevel models for each of the CMRFs analysed in this study are presented in Tables 5, 6, 7, 8, 9, 10 and 11. After adjusting for the covariates, all seven CMRFs were found to be significantly associated with area-level IRSD in the study region. For all but one variable the associations were positive (i.e. increased with area-level disadvantage). TC was the exception; being inversely associated with area-level disadvantage, with the most disadvantaged quintile (Q1) displaying the lowest odds for *higher risk* test results. Among the covariates, there was no significant association between gender and *higher risk* test results of eGFR or BMI. It was also noted that the odds of *higher risk* eGFR tests results accelerated with increasing age group, and the 80+ age group demonstrated a very high odds of being identified with a *higher risk* eGFR tests result in the study region.

The overall comparisons of model random effects are presented in Table 12. Reductions in the AIC values were observed among all CMRFs from the null model (M1) to the final model (M4) indicating a better fit for the final models. In the unadjusted null models, *higher risk* test results of eGFR demonstrated the most area-level variance (0.189) and TC the least (0.026). Adjusting the CMRFs for age and sex initially increased the τ^2^ of M2 for FBSL (PCV =  + 1.88%), HbA1c (PCV =  + 3.02%), HDL (PCV =  + 15.25%) and BMI (PCV =  + 1.48%). The τ^2^ was reduced in the final model among all CMRFs compared with the null models.

**Table 12:** Summary model fit values and comparison of the multilevel models.

	FBSL	HbA1c	TC	HDL	ACR	eGFR	Obesity
**Model 0: Single level model, adjusted for age and sex**							
AIC	103,645	49,897	227,464	123,277	16,596	115,340	242,064
AUC	0.70	0.56	0.63	0.67	0.65	0.88	0.56
**Model 1: Null Model, at level 1**							
AIC	111,022.8	50,114.5	235,931.6	130,649.7	17,130.0	167,164.8	242,793.2
${\tau }^{2}$	0.101	0.103	0.026	0.071	0.092	0.189	0.115
ICC (%)	3.0	3.0	0.8	2.1	2.7	5.4	3.4
MOR	1.35	1.36	1.16	1.29	1.34	1.51	1.38
AUC	0.61	0.63	0.56	0.60	0.70	0.64	0.60
**Model 2: Age and sex adjusted model, at level 1**							
AIC	103,066.2	49,690.2	227,254.6	122,700.0	16,585.2	115,257.1	239,122.6
${\tau }^{2}$	0.103	0.106	0.020	0.081	0.073	0.024	0.117
ICC (%)	3.0	3.1	0.6	2.4	2.2	0.7	3.4
MOR	1.36	1.36	1.14	1.31	1.30	1.16	1.39
AUC	0.73	0.64	0.64	0.71	0.69	0.89	0.63
AUC change†	+ 0.03	+ 0.08	+ 0.01	+ 0.04	+ 0.04	+ 0.01	+ 0.07
PCV	+ 1.88%	+ 3.02%	− 21.76%	+ 15.25%	− 20.53%	− 87.26%	+ 1.48%
**Model 3: IRSD adjusted model, at level 1**							
AIC	110,552.5	49,875.3	235,795.4	130,294.3	17,053.0	166,930.0	242,443.7
${\tau }^{2}$	0.034	0.047	0.018	0.030	0.044	0.138	0.071
ICC (%)	1.0	1.4	0.6	0.9	1.3	4.0	2.1
MOR	1.19	1.23	1.14	1.18	1.22	1.43	1.29
AUC	0.60	0.61	0.56	0.59	0.62	0.63	0.60
PCV	− 66.41%	− 54.82%	− 27.81%	− 58.05%	− 52.88%	− 26.84%	− 38.76%
**Model 4: Age, sex and IRSD adjusted final model, at level 1**							
AIC	102,689.6	49,453.3	227,199.2	122,328.3	16,527.2	115,125.7	238,748.4
${\tau }^{2}$	0.044	0.049	0.017	0.034	0.036	0.014	0.069
ICC (%)	1.3	1.5	0.5	1.0	1.1	0.4	2.0
MOR	1.22	1.24	1.13	1.19	1.20	1.12	1.28
AUC	0.72	0.63	0.64	0.70	0.67	0.88	0.62
PCV	− 56.33%	− 51.91%	− 33.27%	− 51.37%	− 61.19%	− 92.79%	− 40.30%

^†^Change in relation to M0; τ^2^—Variance; AIC—Akaike Information Criterion; AUC—Area under the receiver operating characteristic curve; ICC—Intra-cluster Correlation Coefficients; MOR—Median Odds Ratios; PCV—proportional change in variance (in relation to Model 1 s).

The Akaike Information Criterion (AIC) was used to evaluate model fit. The derived multilevel models were compared for: area-level variance (${\tau }^{2})$ at SA1 (level 2) level; proportional change in variance (PCV); Intra-cluster Correlation Coefficients (ICC); Median Odds Ratios (MORs); Area under the receiver operating characteristic (AUC) curve; and the change in AUC.

The ICCs of the unadjusted models ranged between 0.8% in high TC to 5.4% in low eGFR. Inclusion of IRSD after adjusting for age and sex had reduced the ICCs of all CMRFs in the final models, which ranged between 0.4% in low eGFR to 2.0% in obesity test results. The ICCs of the final models were low and suggest very limited area-level contextual effects. The AUC changes in model 2 and MORs of the final model support these findings.

**Figure 2:**
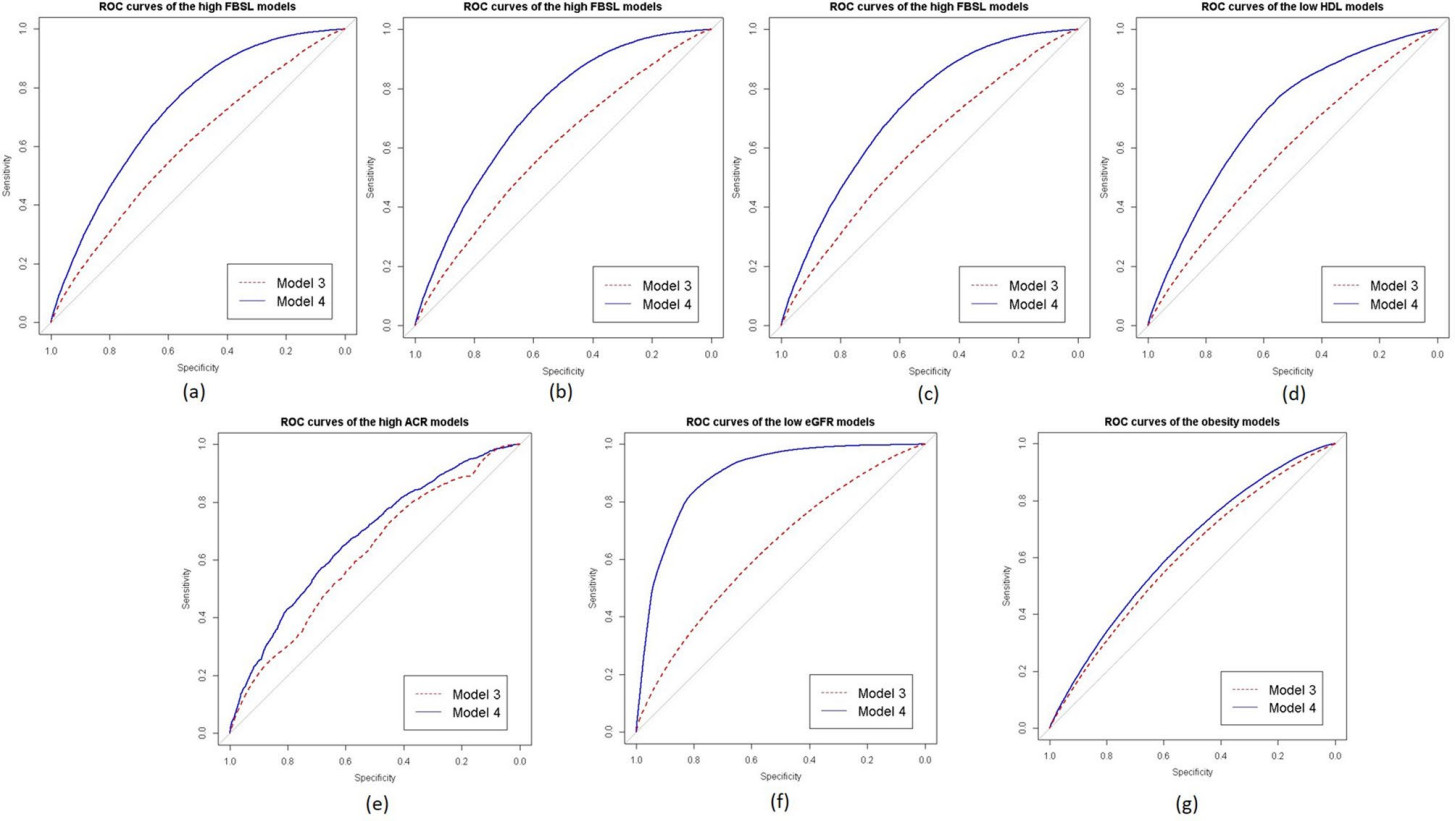
ROC curves of the fitted models (Model 3s and Model 4s) of CMRFs for comparison: (**a**) FBSL models; (**b**) HbA1c models; (**c**) TC models; (**d**) HDL models; (**e**) ACR models; (**f**) FBSL models; (**g**) obesity models; Model 3s—CMRFs adjusted only for IRSD quintiles of areas; Model 4s—Final models of CMRFs adjusted for age + sex + IRSD quintiles of area.

Figure 2 provides a comparison of the ROC curves of the fitted models. Model 4 s (age + sex + IRSD adjusted models) and models 3 s (IRSD adjusted models) were chosen for the ROC curve plotting for comparative purpose. The predicted outcomes in the CMRFs plots are for the reference individual, i.e., individuals residing in the least disadvantaged areas (model 3) + female + age group 18–29 years (Model 4). A model curve closer to the top left corner of the subfigures indicate a better predictive accuracy of the model. The single measure summary of the ROC curves, AUCs of the final models ranged 0.62–0.88. The highest AUC value was observed for the final model of low eGFR. The AUC changes of model 2 s in relation to M0s ranged 0.01–0.08, which reconfirm the contextual findings of ICCs that the general contextual effects observed in the models were minimal.

The proportions of the geographic variance in CMRFs contributed by IRSD were estimated through the PCV between M2 and M4. Adjusting the models for IRSD and individual-level variables explained a maximum 92.79% of the variance expressed by the null model of eGFR, reducing the ICC from 5.4 to 0.4%. The changes were least among the adjusted models of TC, with a marginal reduction of ICC from 0.8% to 0.5%. Thus, in the final models, the proportional reduction in variance was the largest for eGFR (PCV = 92.79%) and the least for TC (PCV = 33.27%).

The identified specific contribution of IRSD in the geographic variation of CMRF was the highest among the geographic variance of *higher risk* findings of HDL tests (57.8%), which was closely followed by FBSL (57.14%); HbA1c (53.31%); and ACR (51.17%) test results. The contribution of IRSD was comparatively lower among the geographic variance of the *higher risk* findings of eGFR (41.75%); BMI (41.06%); and TC (14.71%) test results, though not the least. Even though these specific proportions are large, it should be noted that it actually explained a lot of very little (i.e., variance of 0.01–0.07).

## Discussion

The study reports on the influence of areas on *higher risk* CMRF distribution and quantifies the specific proportion of geographic variance explained by IRSD. The work adds to the very few studies which consider multiple CMRF variables within the same region, or which are based on population derived data over extended years^16,17,20,29,31,32^,and reports on both single and multilevel analyses^38,55^. The results present both the measures of association and area-level variance based on multilevel logistic regression analyses^36^. The findings of the study add to the existing evidence and discussion regarding the relevance of individual versus area-level interventions for the prevention and control of CMRFs.

We found consistent evidence for the association between area-level disadvantage and seven CMRFs among adult health service using residents of the Illawarra-Shoalhaven region in NSW Australia. In adjusted models, the odds of a *higher risk* finding increased with increase in area-level disadvantage among all CMRFs excepting TC, which showed an inverse pattern of association with increase in area-level disadvantage. Thus, in the final models we observed that, over and above individual age and sex, living in a disadvantaged neighbourhood proportionally increased the individual-level probability of being identified with a *higher risk* CMRF. The findings highlight the importance of including of area-level variables into health risk analyses.

The ICCs of CMRFs in all the models were comparatively small (Table 12) in all the models. In the fully adjusted models, the ICCs were further reduced and ranged between 0.4% and 2.0% in low eGFR and BMI respectively. As per the interpretation framework proposed by Merlo et al., an ICC value less than 10% is indicative of very little geographic difference^56^. The AUC change of the model 2 s in relation to the single level models (range 0.01–0.08) reconfirm on these findings. However, this has to be interpreted along with the traditional geographic comparisons such as the proportion of the individuals who are affected with *higher risk* CMRF outcomes. Therefore, a small geographic difference with uniformly higher, medium, or lower proportion of affected individuals indicates homogeneity of the *higher risk* CMRF findings within their geographic units^56^. Such a situation would call for balanced universal approaches to prevent and control the *higher risk* CMRFs, with a proportional focus to the need and disadvantage level of affected populations^57,58^. However, it is also worth noting that when the exposure to an agent is homogenic in a community, the traditional epidemiological methods are not very helpful in identifying their markers of susceptibility^59^.

Our results confirm, and are comparable with, associations between area-level disadvantage and CMRFs reported in previous studies^3–23^, and extends their findings. The results primarily confirm the geographic variation of CMRFs and associations with area level disadvantage, as reported in previous studies. Further, the study provides means to compare this association which were observed consistently with a range of multiple CMRFs analysed in this study. The study extends on previous reports by differentiating the individual and area-level contributors to the exhibited geographic variance of CMRFs. And most importantly, the general contextual effect and the specific contributions of IRSD on the geographic variance of multiple CMRFs were identified, which is unique in the literature and highly informative for health care service commissioning.

The TC test results often stood apart from the major findings of this study, demonstrating inverse associations with IRSD. However, this was not reflected in the HDL findings, even though both are components of the lipid profile in an individual. This raises the possibility of a medication effect on TC in these areas, where the lipid lowering drugs have a less consistent effect in raising HDL than in lowering TC^60^. Other factors associated with the *higher risk* HDL test results may include uncontrolled diabetes^61^^,^ smoking^62^^,^ sedentary life style^63,64^, obesity^65^^,^ and poor diet quality^66,67^. However the reason for the inverse association demonstrated by TC test results are not clearly established within the current study results and requires further research to explore possible individual and area-level contributions.

The study has to be considered within its limitations. Primarily, the cross sectional nature of analyses adopted in this study do not yield support for any causal relationships. In addition, the non-linear and time varying effects of covariates analysed in this study restrict generalisability of their findings though very informative for regional health care service commissioning. Secondly, the IRSD quintiles included as the key explanatory variable represent relative disadvantage in an area and have limitations intrinsic to aggregate measures. Thirdly, it should be noted that the data used in this study are extracted from people already utilising the health care service facilities in the area. Fourthly , the readers should be mindful that the variance reported in this study are attributable to (1) individual level factors (age, sex) analysed at the area-level, (2) area-level contextual influences (IRSD), and (3) other individual and area-level characteristics not considered in this study. However, further individual-level data extractions or collections are not possible with this study’s dataset as the de-identification process precludes the inclusion of any further individual level data. Other individual and area-level factors not considered in this study could include: individual-level SES^68^, type of neighbourhood food outlets^69–72^, poor physical activity resources^73,74^, residential density and service availability^75^. Finally, the assumptions of the standard multilevel logistic regression modelling methods adopted in this study would not be able to account for the autocorrelation of the area-level residuals (if any) of the models. Expected shortcomings due to this could be an overestimation of random effects in our models^76^. However, any such effects were observed to be very marginal in our results as the random effect estimates are already at their lower limits. While acknowledging this limitation, we believe the effects of this are not critical in our results. Hybrid models which provide more precise estimates of random effects are becoming increasingly available with advances in computational technologies^77^. However, they would not be directly applicable to our data sets, mainly due to the non-availability of location specific data at individual-level in our study data.

Notwithstanding these limitations, the study is unique in that it analysed a range of CMRFs across a widely dispersed population and included both rural and urban residents. In addition, the study used six years (year 2012–2017) of CMRF tests data from the region in the hierarchical multilevel analyses. The findings of the study indicate that those residing in the most disadvantaged areas are more likely to be identified with *higher risk* CMRFs than those in lower disadvantage areas. However, the low ICC, AUC change and MOR values of the area-level models do not support for contextual approaches. Rather, the findings of the study support a proportionate universalism approach in which health resources are made universally available but proportional to the need and disadvantage level of the affected population^57,58^.

## Conclusion

The study demonstrates that in the Illawarra Shoalhaven region of Australia, people residing in socioeconomically disadvantaged areas have a higher probability of being identified with *higher risk* CMRFs across a range of factors. The low general contextual effects of the areas suggest for universal intervention for the prevention and control of CMRFs in this study region, but proportional to the need and disadvantage level. The patterns were consistent across the six CMRFs analysed in this study; and comparable with similar studies reported nationally and globally. Based on our findings, we recommend further area-level research to discern the role of other contextual factors not analysed in this study especially the area-level access to health care services to determine its existing role and adequacy^78^, and evidence based universal interventions for the prevention and control of CMRFs but proportionate to the priority level of the populations based on area-level disadvantage.

## Data Availability

Access to, and use of, Southern IML Research (SIMLR) Study data are subject to a License Agreement—Provision of Data (LA) between Southern IML Pathology Pty Ltd (Data Owner) and The University of Wollongong (License Holder), and a Data Access Agreement (DAA) between the License Holder and researchers (Data Users). This process is facilitated by the Illawarra Health and Medical Research Institute (IHMRI) (Data Custodian) through the Southern IML Research Study—Cohort Management Committee (SIMLR—CMC). The Data License does not allow for “public access” to data; however, researcher may access to SIMLR Study data subject to approval by the SIMLR—CMC and an appropriately constituted Australian Human Research Ethics Committee (HREC) as defined in the National Health and Medical Research Council’s National Statement on Ethical Conduct in Human Research (2007) (available from https://www.nhmrc.gov.au/about-us/publications/national-statement-ethical-conduct-human-research-2007-updated-2018). The Data License requires at least one of the research team be affiliated with IHMRI. SIMLR—CMC contact details are: C/o-Associate Professor Kathryn Weston; Southern IML Research Study—Cohort Management Committee; Illawarra Health and Medical Research Institute; Building 32, University of Wollongong, Northfields Avenue, Wollongong NSW 2522, Australia; Phone  +61 2 4221 4333; Email: info@ihmri.org.au; Web Link: https://www.ihmri.org.au/research-projects/simlr-cohort-study/.
